# Crystal structure and Hirshfeld surface analysis of (*Z*)-6-[(2-hy­droxy-4-methyl­anilino)­methyl­idene]-4-methyl­cyclo­hexa-2,4-dien-1-one

**DOI:** 10.1107/S2056989019006583

**Published:** 2019-05-14

**Authors:** Sevgi Kansiz, Alev Sema Aydin, Necmi Dege, Erbil Agar, Igor O. Fritsky

**Affiliations:** a Ondokuz Mayıs University, Faculty of Arts and Sciences, Department of Physics, 55139, Kurupelit, Samsun, Turkey; b Ondokuz Mayıs University, Faculty of Arts and Sciences, Department of Physics, 55139, Samsun, Turkey; c Ondokuz Mayıs University, Faculty of Arts and Sciences, Department of Chemistry, 55139, Samsun, Turkey; d Taras Shevchenko National University of Kyiv, Department of Chemistry, 64, Vladimirska Str., Kiev 01601, Ukraine

**Keywords:** crystal structure, Schiff base, hydrogen bonding, Hirshfeld surface analysis

## Abstract

The title compound, C_15_H_15_NO_2_, is a Schiff base that exists in the keto–enamine tautomeric form and adopts a *Z* configuration. The mol­ecule is almost planar, with the two phenyl rings twisted relative to each other by 9.60 (18)°. There is an intra­molecular N—H⋯O hydrogen bond present forming an *S*(6) ring motif. In the crystal, pairs of O—H⋯O hydrogen bonds link adjacent mol­ecules into inversion dimers with an *R* ^2^_2_(18) ring motif. The dimers are linked by very weak π–π inter­actions, forming layers parallel to (01). Hirshfeld surface analysis, two-dimensional fingerprint plots and the mol­ecular electrostatic potential surfaces were used to analyse the inter­molecular inter­actions, indicating that the most important contributions for the crystal packing are from H⋯H (55.2%), C⋯H/H⋯C (22.3%) and O⋯H/H⋯O (13.6%) inter­actions.

## Abstract 2

In the crystal structure of the title compound, the mol­ecules are linked by pairs of O—H⋯O hydrogen bonds, forming inversion dimers with an 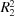
(18) ring motif.

## Chemical context   

Schiff bases contain the azomethine moiety (–*R*CH=N–*R*′) and are prepared by condensation reactions between amines and active carbonyl compounds (Schiff, 1864 ▸). In the majority of cases, the synthesis involves an aromatic amine and an aldehyde (Schiff *et al.*, 1881 ▸). Schiff bases are very important for production of chemical specialties such as pharmaceuticals including anti­biotics, and of anti­allergic, anti­tumor, anti­fungal, anti­bacterial, anti­malarial or anti­viral drugs. Schiff bases are also employed as catalyst carriers (Grigoras *et al.*, 2001 ▸), thermo-stable materials (Vančo *et al.*, 2004 ▸), metal–cation complexing agents or in biological systems (Taggi *et al.*, 2002 ▸). Schiff bases containing phenol indicate two possible tautomeric forms, *viz*. phenol–imine and keto–enamine.
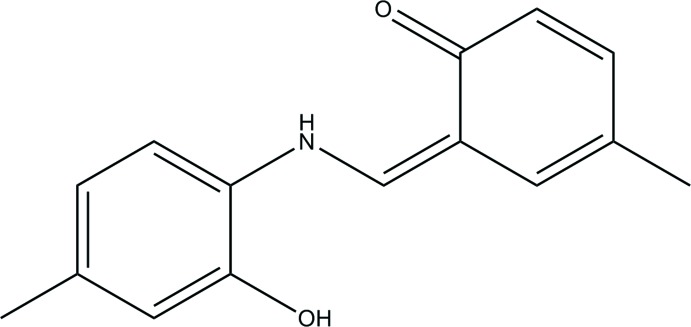


In the current study, a new Schiff base, (*Z*)-6-{[(2-hy­droxy-4-methyl­phen­yl)amino]­methyl­idene}-4-methyl­cyclo­hexa-2,4-dien-1-one, was obtained in crystalline form from the reaction of 2-amino-5-methyl­phenol with 2-hy­droxy-5-methyl­benz­aldehyde. We report here its synthesis conditions and the mol­ecular and crystal structures, supplemented by Hirshfeld surface analysis.

## Structural commentary   

**Figure 1:**
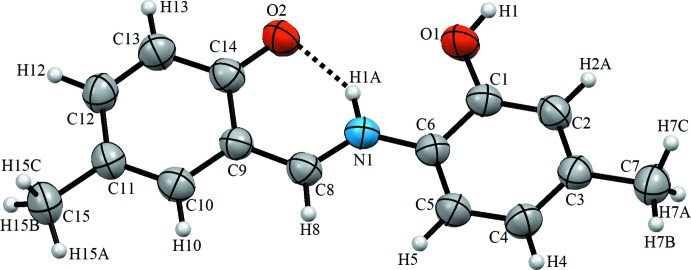
The mol­ecular structure of the title compound, with displacement ellipsoids drawn at the 40% probability level. Dashed lines denote the intra­molecular N—H⋯O hydrogen bond (Table 1 ▸) forming an *S*(6) ring motif.

The mol­ecular structure of the title compound is illustrated in Fig. 1 ▸. The asymmetric unit comprises one mol­ecule that adopts the keto–enamine tautomeric form, *i.e.* the H atom is located at the amine functionality (N1). The mol­ecule is almost planar, with an r.m.s. deviation of 0.1061 Å for the complete mol­ecule except the H atoms [largest deviation 0.176 (3) Å for C8]. The two phenyl rings (C1–C6 and C9–C14) are inclined by 9.60 (18)°. The C1—O1 bond length [1.356 (3) Å] to the hy­droxy group is in the normal range, while the C14=O2 bond length is comparatively elongated [1.302 (4) Å] due to the involvement of the carbonyl O atom in an intra­molecular N—H⋯O hydrogen bond, forming an *S*(6) ring motif. The C6—N1 and C8=N1 bond lengths are 1.404 (4) and 1.310 (4) Å, respectively. Overall, the bond lengths in the title structure compare well with those of other keto–enamine tautomers known from the literature (see: *Database Survey*).

## Supra­molecular features   

**Figure 2:**
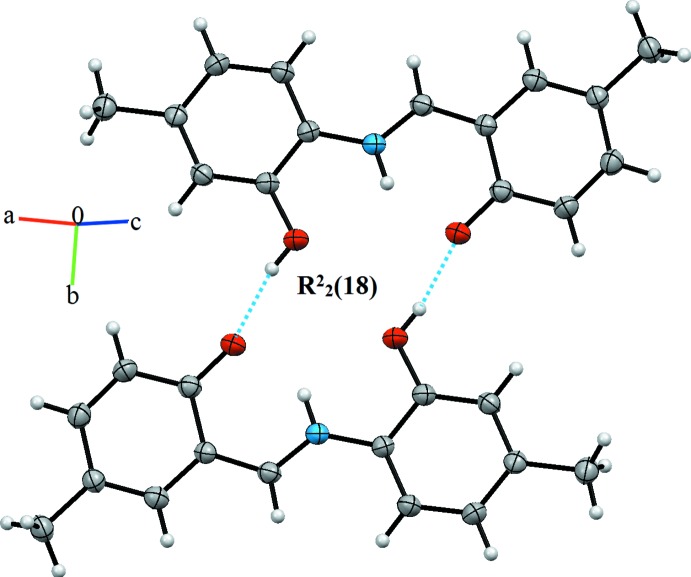
A view of the crystal packing of the title compound. Dashed lines denote the inter­molecular O—H⋯O hydrogen bonds (Table 1 ▸) forming an inversion dimer with an 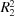
(18) ring motif.

The mol­ecules are linked by mutual O—H⋯O hydrogen bonds forming pairs of inversion dimers with an 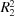
(18) ring motif (Table 1 ▸, Figs. 2 ▸ and 3 ▸). The dimers are linked by very weak π-stacking inter­actions [*Cg*1⋯*Cg*2 = 4.721 (2) Å; *Cg*1 and *Cg*2 are the centroids of the C1–C6 and C9–C14 rings, respectively], forming layers parallel to (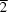
01).

## Database survey   

A search of the Cambridge Structural Database (CSD, version 5.40, update November 2018; Groom *et al.*, 2016 ▸) for the (*E*)-2-[(2-hy­droxy­phenyl­iminio)meth­yl]phenolate fragment revealed 25 hits where this fragment adopts the keto–enamine tautomeric form. Nearly all bond lengths in the title structure are the same within standard uncertainties as the corresponding bond lengths in the structures of 2,4-di­chloro-6-{[(2-meth­oxy­phen­yl)iminio]meth­yl}phenolate hydrate (VUYFEC; Tsuchimoto *et al.*, 2016 ▸), 2-{(*E*)-[(2-hy­droxy­phen­yl)iminio]meth­yl}-4-methyl­phenolate (XULSOO; Shalini *et al.*, 2015 ▸), (*E*)-4-hy­droxy-2-[(2-hy­droxy­phen­yl)iminiometh­yl]phenolate (QUYGOH; Eltayeb *et al.*, 2010 ▸) or 2-{(*E*)-[(2-hy­droxy-5-methyl­phen­yl)iminio]meth­yl}-4-(tri­fluoro­meth­oxy)phenolate (QAJYUX; Karadağ *et al.*, 2011 ▸). For example, in the structures of these typical keto–enamine tautomers, the corresponding C14=O2 and C8—C9 bond lengths are in the ranges 1.279–1.316 Å and 1.410–1.427 Å, respectively. It is likely that the inter­molecular O—H⋯O hydrogen bond, where the keto O atom acts as an hydrogen-bond acceptor, is an important prerequisite for the tautomeric shift toward the keto–enamine form. In fact, in all 25 structures of the keto–enamine tautomers, hydrogen bonds of this type are observed.

## Hirshfeld surface analysis   

**Figure 4:**
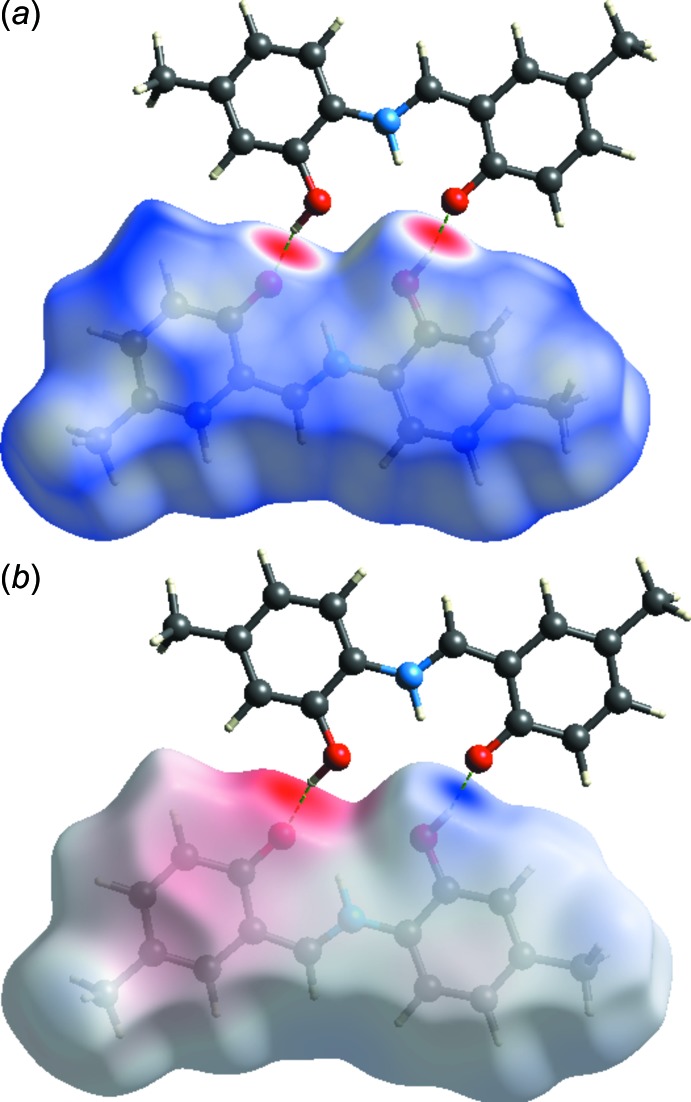
(*a*) The Hirshfeld surface mapped over *d*_norm_, and (*b*) the mol­ecular electrostatic potential surface showing the O—H⋯O inter­actions.

A Hirshfeld surface analysis (Spackman & Jayatilaka, 2009 ▸) and the associated two-dimensional fingerprint plots (McKinnon *et al.*, 2007 ▸) were performed with *CrystalExplorer17* (Turner *et al.*, 2017 ▸) for specifying the inter­molecular inter­actions in the title compound. Fig. 4 ▸ *a* illustrates the Hirshfeld surface mapped over *d*_norm_. The red spots highlight the inter­atomic contacts included in O—H⋯O hydrogen bonding. The three-dimensional *d*_norm_ surfaces were plotted with a colour scale of −0.7370 to 1.3366 Å with a standard (high) surface resolution. Fig. 4 ▸ *b* shows the mol­ecular electrostatic potential plotted over the three-dimensional Hirshfeld surface using the STO-3G basis set in the range −0.0975 to 0.2197 a.u. within the Hartree–Fock level of theory. The O—H⋯O hydrogen-bond donors and acceptors are shown as blue and red areas around the atoms related with positive (hydrogen-bond donors) and negative (hydrogen-bond acceptors) electrostatic potentials, respectively.

**Figure 5:**
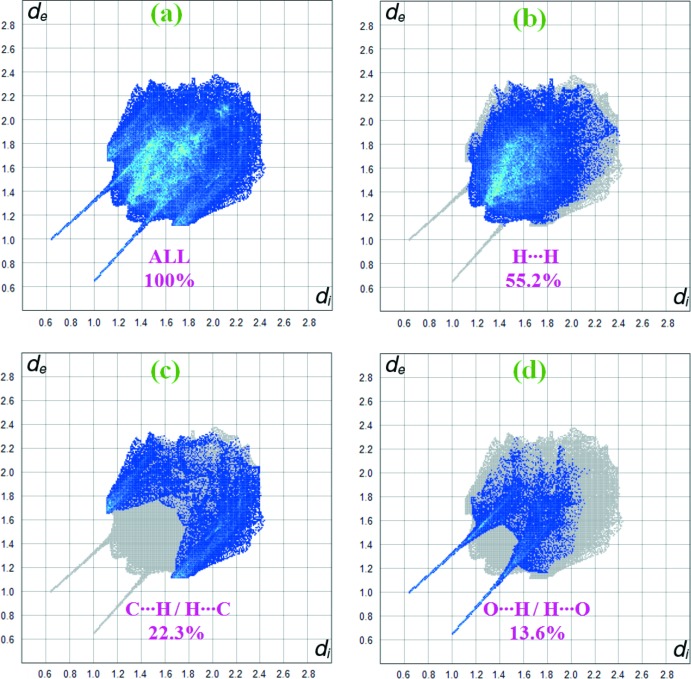
Two-dimensional fingerprint plots for the title compound giving the relative contribution of atom pairs to the Hirshfeld surface.

Fig. 5 ▸ *a* shows the two-dimensional fingerprint of the sum of all contacts contributing to the Hirshfeld surface indicated in normal mode. Fig. 5 ▸ *b* illustrates the two-dimensional fingerprint of (*d*_i_, *d*_e_) points related to H⋯H contacts that represent a 55.2% contribution in the title structure. In Fig. 5 ▸ *c*, two symmetrical wings on the left and right sides indicate C⋯H/H⋯C inter­actions with a contribution of 22.3%. Furthermore, there are O⋯H/H⋯O (13.6%; Fig. 5 ▸ *d*), C⋯C (4.9%) and C⋯N/N⋯C (2.6%) contacts.

**Figure 6:**
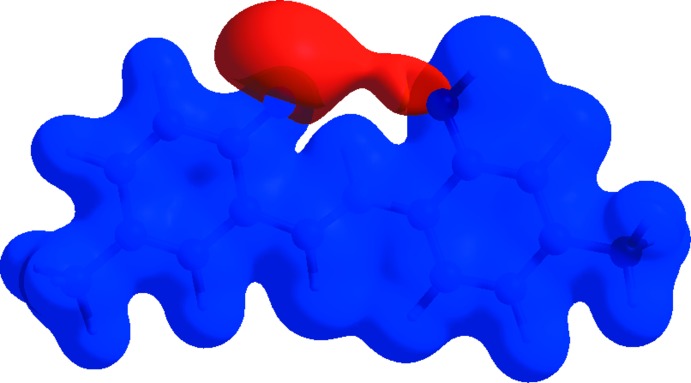
A view of the mol­ecular electrostatic potential, in the range −0.0500 to 0.0500 a.u. calculated using the STO-3 G basis set in the range −0.050 to 0.050 a.u. within the Hartree–Fock level of theory.

Fig. 6 ▸ shows the mol­ecular electrostatic potential surface generated using the STO-3G basis set in the range −0.050 to 0.050 a.u. within the Hartree–Fock level of theory. The blue and red regions are associated with positive and negative mol­ecular electrostatic potentials and represent the donor and acceptor groups, respectively, in hydrogen bonding.

## Synthesis and crystallization   

The title compound was prepared by refluxing a mixture of 2-hy­droxy-5-methyl­benzaldehyde (34.0 mg, 0.25 mmol) in ethanol (15 ml) and 2-amino-5-methyl­phenol (30.8 mg, 0.25 mmol) in ethanol (15 ml) for 5 h. Single crystals of the title compound for X-ray analysis were obtained by slow evaporation of an ethanol solution (yield 65%, m.p. 446–448 K).

## Refinement   

Crystal data, data collection and structure refinement details are summarized in Table 2 ▸. The O- and N-bound H atoms were located in a difference-Fourier map and refined with O—H = 0.82 Å and N—H = 0.85 Å, and with *U*_iso_(H) = 1.5*U*_eq_(N,O). The C-bound H atoms were positioned geometrically and refined using a riding model with C—H = 0.93 and *U*_iso_(H) = 1.2*U*_eq_(C) for aromatic H atoms, and with C—H = 0.96 Å and *U*_iso_(H) = 1.5*U*_eq_(C) for methyl H atoms.

## Supplementary Material

Crystal structure: contains datablock(s) I. DOI: 10.1107/S2056989019006583/wm5504sup1.cif

Structure factors: contains datablock(s) I. DOI: 10.1107/S2056989019006583/wm5504Isup2.hkl

CCDC reference: 1902148

Additional supporting information:  crystallographic information; 3D view; checkCIF report

## Figures and Tables

**Figure 3:**
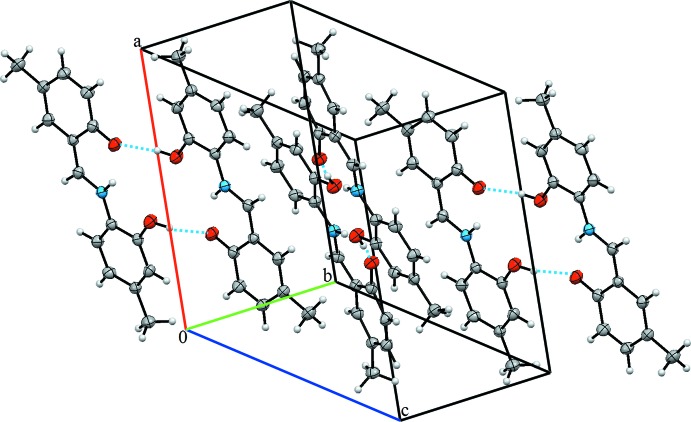
The crystal packing of the title compound.

**Table 1:** Hydrogen-bond geometry (Å, °)

*D*—H⋯*A*	*D*—H	H⋯*A*	*D*⋯*A*	*D*—H⋯*A*
O1—H1⋯O2^i^	0.82	1.82	2.627 (3)	168
N1—H1*A*⋯O2	0.87 (4)	1.83 (4)	2.585 (4)	144 (3)

Symmetry code: (i) 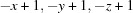.

**Table 2:** Experimental details

Crystal data	
Chemical formula	C_15_H_15_NO_2_
*M*_r_	241.28
Crystal system, space group	Monoclinic, *P*2_1_/*c*
Temperature (K)	296
*a*, *b*, *c* (Å)	11.3954 (19), 11.746 (2), 10.3067 (17)
β (°)	115.940 (12)
*V* (Å^3^)	1240.6 (4)
*Z*	4
Radiation type	Mo *K*α
μ (mm^−1^)	0.09
Crystal size (mm)	0.57 × 0.50 × 0.44

Data collection	
Diffractometer	Stoe *IPDS* 2
Absorption correction	Integration (*X-RED32*; Stoe & Cie, 2002 ▸)
*T*_min_, *T*_max_	0.962, 0.975
No. of measured, independent and observed [*I* > 2σ(*I*)] reflections	6997, 2417, 1261
*R*_int_	0.061
(sin θ/λ)_max_ (Å^−1^)	0.617

Refinement	
*R*[*F* ^2^ > 2σ(*F* ^2^)], *wR*(*F* ^2^), *S*	0.064, 0.156, 0.99
No. of reflections	2417
No. of parameters	171
H-atom treatment	H atoms treated by a mixture of independent and constrained refinement
Δρ_max_, Δρ_min_ (e Å^−3^)	0.16, −0.14

Computer programs: *X-AREA* and *X-RED* (Stoe & Cie, 2002 ▸), *SHELXT2017* (Sheldrick, 2015*a* ▸), *SHELXL2018* (Sheldrick, 2015*b* ▸), *WinGX* (Farrugia, 2012 ▸), *PLATON* (Spek, 2009 ▸) and *publCIF* (Westrip, 2010 ▸).

## supplementary crystallographic information

### Crystal data

**Table:**

C_15_H_15_NO_2_	*F*(000) = 512
*M**_r_* = 241.28	*D*_x_ = 1.292 Mg m^−^^3^
Monoclinic, *P*2_1_/*c*	Mo *K*α radiation, λ = 0.71073 Å
*a* = 11.3954 (19) Å	Cell parameters from 15101 reflections
*b* = 11.746 (2) Å	θ = 2.8–30.9°
*c* = 10.3067 (17) Å	µ = 0.09 mm^−^^1^
β = 115.940 (12)°	*T* = 296 K
*V* = 1240.6 (4) Å^3^	Prism, red
*Z* = 4	0.57 × 0.50 × 0.44 mm

### Data collection

**Table:**

Stoe IPDS 2 diffractometer	2417 independent reflections
Radiation source: sealed X-ray tube, 12 x 0.4 mm long-fine focus	1261 reflections with *I* > 2σ(*I*)
Detector resolution: 6.67 pixels mm^-1^	*R*_int_ = 0.061
rotation method scans	θ_max_ = 26.0°, θ_min_ = 2.8°
Absorption correction: integration (X-RED32; Stoe & Cie, 2002)	*h* = −14→13
*T*_min_ = 0.962, *T*_max_ = 0.975	*k* = −14→11
6997 measured reflections	*l* = −12→12

### Refinement

**Table:**

Refinement on *F*^2^	Hydrogen site location: mixed
Least-squares matrix: full	H atoms treated by a mixture of independent and constrained refinement
*R*[*F*^2^ > 2σ(*F*^2^)] = 0.064	*w* = 1/[σ^2^(*F*_o_^2^) + (0.0717*P*)^2^] where *P* = (*F*_o_^2^ + 2*F*_c_^2^)/3
*wR*(*F*^2^) = 0.156	(Δ/σ)_max_ < 0.001
*S* = 0.99	Δρ_max_ = 0.16 e Å^−^^3^
2417 reflections	Δρ_min_ = −0.14 e Å^−^^3^
171 parameters	Extinction correction: SHELXL2017 (Sheldrick, 2015b), Fc^*^=kFc[1+0.001xFc^2^λ^3^/sin(2θ)]^-1/4^
0 restraints	Extinction coefficient: 0.007 (2)

### Special details

**Table:**

Geometry. All esds (except the esd in the dihedral angle between two l.s. planes) are estimated using the full covariance matrix. The cell esds are taken into account individually in the estimation of esds in distances, angles and torsion angles; correlations between esds in cell parameters are only used when they are defined by crystal symmetry. An approximate (isotropic) treatment of cell esds is used for estimating esds involving l.s. planes.

### Fractional atomic coordinates and isotropic or equivalent isotropic displacement parameters (Å^2^)

**Table:**

	*x*	*y*	*z*	*U*_iso_*/*U*_eq_	
O1	0.3892 (2)	0.58075 (18)	0.4721 (3)	0.0775 (7)	
H1	0.379271	0.539357	0.530605	0.116*	
O2	0.6238 (2)	0.57505 (18)	0.3518 (2)	0.0735 (7)	
N1	0.4922 (3)	0.7437 (3)	0.3822 (3)	0.0581 (7)	
C9	0.6549 (3)	0.7665 (2)	0.2975 (3)	0.0544 (7)	
C1	0.3454 (3)	0.6868 (3)	0.4791 (3)	0.0563 (7)	
C8	0.5565 (3)	0.8082 (3)	0.3317 (3)	0.0599 (8)	
H8	0.536018	0.885293	0.317983	0.072*	
C6	0.3978 (3)	0.7746 (2)	0.4288 (3)	0.0546 (7)	
C2	0.2544 (3)	0.7113 (3)	0.5301 (3)	0.0613 (8)	
H2A	0.219587	0.652120	0.562261	0.074*	
C3	0.2140 (3)	0.8211 (3)	0.5343 (3)	0.0592 (8)	
C11	0.8287 (3)	0.8104 (3)	0.2270 (3)	0.0603 (8)	
C14	0.6898 (3)	0.6484 (3)	0.3155 (3)	0.0599 (8)	
C10	0.7254 (3)	0.8436 (3)	0.2515 (3)	0.0628 (8)	
H10	0.700067	0.919577	0.237683	0.075*	
C5	0.3571 (3)	0.8851 (3)	0.4324 (3)	0.0642 (8)	
H5	0.390952	0.944514	0.399559	0.077*	
C4	0.2668 (3)	0.9080 (3)	0.4843 (3)	0.0670 (8)	
H4	0.240644	0.982737	0.485967	0.080*	
C12	0.8638 (3)	0.6949 (3)	0.2488 (3)	0.0681 (9)	
H12	0.934719	0.670249	0.234291	0.082*	
C13	0.7972 (3)	0.6170 (3)	0.2906 (3)	0.0688 (9)	
H13	0.823971	0.541368	0.302935	0.083*	
C7	0.1151 (3)	0.8461 (3)	0.5909 (3)	0.0762 (10)	
H7A	0.031563	0.816866	0.524800	0.114*	
H7B	0.109049	0.926922	0.600598	0.114*	
H7C	0.141812	0.810509	0.683322	0.114*	
C15	0.9064 (3)	0.8936 (3)	0.1829 (4)	0.0769 (10)	
H15A	0.861304	0.965184	0.157474	0.115*	
H15B	0.916047	0.863810	0.101333	0.115*	
H15C	0.990962	0.904493	0.261813	0.115*	
H1A	0.516 (4)	0.673 (4)	0.387 (4)	0.088 (12)*	

### Atomic displacement parameters (Å^2^)

**Table:**

	*U*^11^	*U*^22^	*U*^33^	*U*^12^	*U*^13^	*U*^23^
O1	0.1026 (18)	0.0475 (13)	0.1056 (17)	0.0073 (13)	0.0671 (15)	0.0093 (11)
O2	0.0894 (16)	0.0526 (13)	0.0949 (14)	−0.0002 (12)	0.0555 (13)	0.0079 (11)
N1	0.0611 (17)	0.0488 (17)	0.0674 (15)	0.0035 (14)	0.0308 (13)	0.0064 (12)
C9	0.0583 (18)	0.0501 (18)	0.0558 (16)	0.0016 (15)	0.0259 (15)	0.0034 (13)
C1	0.0612 (19)	0.0484 (18)	0.0586 (16)	0.0013 (16)	0.0254 (15)	0.0019 (13)
C8	0.0620 (19)	0.0505 (18)	0.0669 (17)	−0.0019 (16)	0.0281 (15)	0.0050 (14)
C6	0.0550 (18)	0.0498 (18)	0.0602 (16)	0.0033 (15)	0.0262 (14)	0.0031 (13)
C2	0.068 (2)	0.056 (2)	0.0655 (17)	−0.0049 (16)	0.0342 (16)	0.0039 (14)
C3	0.0591 (19)	0.063 (2)	0.0557 (16)	0.0044 (17)	0.0251 (14)	0.0027 (14)
C11	0.063 (2)	0.063 (2)	0.0601 (16)	−0.0045 (17)	0.0318 (15)	−0.0012 (14)
C14	0.065 (2)	0.0535 (19)	0.0620 (16)	−0.0024 (16)	0.0288 (15)	−0.0002 (14)
C10	0.067 (2)	0.0563 (19)	0.0648 (17)	−0.0033 (17)	0.0283 (16)	0.0005 (14)
C5	0.0658 (19)	0.0512 (19)	0.0800 (19)	−0.0004 (17)	0.0359 (17)	0.0060 (15)
C4	0.072 (2)	0.0526 (19)	0.084 (2)	0.0078 (17)	0.0406 (18)	0.0011 (16)
C12	0.071 (2)	0.069 (2)	0.0735 (19)	0.0016 (18)	0.0400 (17)	−0.0055 (16)
C13	0.082 (2)	0.0532 (19)	0.081 (2)	0.0030 (18)	0.0450 (19)	−0.0030 (16)
C7	0.073 (2)	0.085 (3)	0.078 (2)	0.0082 (19)	0.0403 (18)	0.0022 (17)
C15	0.078 (2)	0.081 (3)	0.084 (2)	−0.011 (2)	0.0477 (19)	−0.0013 (18)

### Geometric parameters (Å, º)

**Table:**

O1—C1	1.356 (3)	C11—C10	1.365 (4)
O1—H1	0.8200	C11—C12	1.405 (4)
O2—C14	1.302 (4)	C11—C15	1.516 (4)
N1—C8	1.310 (4)	C14—C13	1.404 (4)
N1—C6	1.404 (4)	C10—H10	0.9300
N1—H1A	0.87 (4)	C5—C4	1.377 (4)
C9—C8	1.403 (4)	C5—H5	0.9300
C9—C10	1.422 (4)	C4—H4	0.9300
C9—C14	1.433 (4)	C12—C13	1.373 (4)
C1—C2	1.383 (4)	C12—H12	0.9300
C1—C6	1.400 (4)	C13—H13	0.9300
C8—H8	0.9300	C7—H7A	0.9600
C6—C5	1.385 (4)	C7—H7B	0.9600
C2—C3	1.377 (4)	C7—H7C	0.9600
C2—H2A	0.9300	C15—H15A	0.9600
C3—C4	1.393 (4)	C15—H15B	0.9600
C3—C7	1.506 (4)	C15—H15C	0.9600
			
C1—O1—H1	109.5	C11—C10—C9	122.6 (3)
C8—N1—C6	129.1 (3)	C11—C10—H10	118.7
C8—N1—H1A	111 (2)	C9—C10—H10	118.7
C6—N1—H1A	120 (2)	C4—C5—C6	120.6 (3)
C8—C9—C10	119.4 (3)	C4—C5—H5	119.7
C8—C9—C14	120.9 (2)	C6—C5—H5	119.7
C10—C9—C14	119.6 (3)	C5—C4—C3	121.2 (3)
O1—C1—C2	124.5 (3)	C5—C4—H4	119.4
O1—C1—C6	115.5 (2)	C3—C4—H4	119.4
C2—C1—C6	120.0 (3)	C13—C12—C11	122.4 (3)
N1—C8—C9	123.0 (3)	C13—C12—H12	118.8
N1—C8—H8	118.5	C11—C12—H12	118.8
C9—C8—H8	118.5	C12—C13—C14	121.9 (3)
C5—C6—C1	118.5 (3)	C12—C13—H13	119.1
C5—C6—N1	124.6 (3)	C14—C13—H13	119.1
C1—C6—N1	116.8 (3)	C3—C7—H7A	109.5
C3—C2—C1	121.6 (3)	C3—C7—H7B	109.5
C3—C2—H2A	119.2	H7A—C7—H7B	109.5
C1—C2—H2A	119.2	C3—C7—H7C	109.5
C2—C3—C4	118.0 (3)	H7A—C7—H7C	109.5
C2—C3—C7	120.8 (3)	H7B—C7—H7C	109.5
C4—C3—C7	121.2 (3)	C11—C15—H15A	109.5
C10—C11—C12	117.0 (3)	C11—C15—H15B	109.5
C10—C11—C15	122.4 (3)	H15A—C15—H15B	109.5
C12—C11—C15	120.5 (3)	C11—C15—H15C	109.5
O2—C14—C13	122.6 (3)	H15A—C15—H15C	109.5
O2—C14—C9	121.0 (3)	H15B—C15—H15C	109.5
C13—C14—C9	116.4 (3)		
			
C6—N1—C8—C9	175.8 (3)	C10—C9—C14—C13	2.3 (4)
C10—C9—C8—N1	−176.2 (3)	C12—C11—C10—C9	0.1 (4)
C14—C9—C8—N1	0.5 (4)	C15—C11—C10—C9	−177.9 (3)
O1—C1—C6—C5	179.8 (3)	C8—C9—C10—C11	174.9 (3)
C2—C1—C6—C5	0.4 (4)	C14—C9—C10—C11	−1.8 (4)
O1—C1—C6—N1	−2.2 (4)	C1—C6—C5—C4	−0.1 (4)
C2—C1—C6—N1	178.4 (3)	N1—C6—C5—C4	−177.9 (3)
C8—N1—C6—C5	−1.7 (5)	C6—C5—C4—C3	0.0 (5)
C8—N1—C6—C1	−179.5 (3)	C2—C3—C4—C5	−0.2 (4)
O1—C1—C2—C3	180.0 (3)	C7—C3—C4—C5	−179.9 (3)
C6—C1—C2—C3	−0.7 (4)	C10—C11—C12—C13	1.0 (5)
C1—C2—C3—C4	0.6 (4)	C15—C11—C12—C13	179.1 (3)
C1—C2—C3—C7	−179.7 (3)	C11—C12—C13—C14	−0.5 (5)
C8—C9—C14—O2	6.1 (4)	O2—C14—C13—C12	178.3 (3)
C10—C9—C14—O2	−177.2 (3)	C9—C14—C13—C12	−1.2 (4)
C8—C9—C14—C13	−174.4 (3)		

### Hydrogen-bond geometry (Å, º)

**Table:**

*D*—H···*A*	*D*—H	H···*A*	*D*···*A*	*D*—H···*A*
O1—H1···O2^i^	0.82	1.82	2.627 (3)	168
N1—H1*A*···O2	0.87 (4)	1.83 (4)	2.585 (4)	144 (3)

Symmetry code: (i) −*x*+1, −*y*+1, −*z*+1.

## References

[bb1] Eltayeb, N. E., Teoh, S. G., Fun, H.-K. & Chantrapromma, S. (2010). *Acta Cryst.* E**66**, o1536–o1537. 10.1107/S1600536810020295 PMC3006709 21587785

[bb2] Farrugia, L. J. (2012). *J. Appl. Cryst.* **45**, 849–854.

[bb3] Grigoras, M., Catanescu, O. & Simonescu, C. I. (2001). *Rev. Roum. Chim.* **46**, 927–939.

[bb4] Groom, C. R., Bruno, I. J., Lightfoot, M. P. & Ward, S. C. (2016). *Acta Cryst.* B**72**, 171–179. 10.1107/S2052520616003954 PMC4822653 27048719

[bb5] Karadağ, A. T., Atalay, Ş. & Genç, H. (2011). *Acta Cryst.* E**67**, o95. 10.1107/S1600536810050579 PMC3050197 21522804

[bb6] McKinnon, J. J., Jayatilaka, D. & Spackman, M. A. (2007). *Chem. Commun.* pp. 3814–3816. 10.1039/b704980c 18217656

[bb7] Schiff, H. (1864). *Ann. Chem. Pharm.* **131**, 118–119.

[bb8] Schiff, H. (1881). *Justus Liebigs Ann. Chem.* **210**, 114–123.

[bb9] Shalini, S., Girija, C. R., Jotani, M. M., Sathish, C. D. & Venkatesha, T. V. (2015). *Acta Cryst.* E**71**, o288. 10.1107/S2056989015006374 PMC4420074 25995908

[bb10] Sheldrick, G. M. (2015*a*). *Acta Cryst.* A**71**, 3–8.

[bb11] Sheldrick, G. M. (2015*b*). *Acta Cryst.* C**71**, 3–8.

[bb12] Spackman, M. A. & Jayatilaka, D. (2009). *CrystEngComm*, **11**, 19–32.

[bb13] Spek, A. L. (2009). *Acta Cryst.* D**65**, 148–155. 10.1107/S090744490804362X PMC2631630 19171970

[bb14] Stoe & Cie (2002). *X-AREA* and *X-RED32*. Stoe & Cie GmbH, Darmstadt, Germany.

[bb15] Taggi, A. E., Hafez, A. M., Wack, H., Young, B., Ferraris, D. & Lectka, T. (2002). *J. Am. Chem. Soc.* **124**, 6626–6635. 10.1021/ja0258226 12047183

[bb16] Tsuchimoto, M., Yoshida, N., Sugimoto, A., Teramoto, N. & Nakajima, K. (2016). *J. Mol. Struct.* **1105**, 152–158.

[bb17] Turner, M. J., MacKinnon, J. J., Wolff, S. K., Grimwood, D. J., Spackman, P. R., Jayatilaka, D. & Spackman, M. A. (2017). *Crystal Explorer Ver. 17.5*. University of Western Australia. http://hirshfeldsurface.net.

[bb18] Vančo, J., Švajlenová, O., Račanská, E. J., Muselík, J. & Valentová, J. (2004). *J. Trace Elem. Med. Biol.* **18**, 155–161. 10.1016/j.jtemb.2004.07.003 15646262

[bb19] Westrip, S. P. (2010). *J. Appl. Cryst.* **43**, 920–925.

